# Implementation of Brief Submaximal Cardiopulmonary Testing in a High-Volume Presurgical Evaluation Clinic: Feasibility Cohort Study

**DOI:** 10.2196/65805

**Published:** 2025-02-17

**Authors:** Zyad James Carr, Daniel Agarkov, Judy Li, Jean Charchaflieh, Andres Brenes-Bastos, Jonah Freund, Jill Zafar, Robert B Schonberger, Paul Heerdt

**Affiliations:** 1 Department of Anesthesiology Yale University New Haven, CT United States; 2 School of Medicine Yale University New Haven, CT United States; 3 Syracuse University Syracuse, NY United States

**Keywords:** preoperative evaluation, submaximal cardiopulmonary exercise test, risk stratification, perioperative medicine, anesthesiology

## Abstract

**Background:**

Precise functional capacity assessment is a critical component for preoperative risk stratification. Brief submaximal cardiopulmonary exercise testing (smCPET) has shown diagnostic utility in various cardiopulmonary conditions.

**Objective:**

This study aims to determine if smCPET could be implemented in a high-volume presurgical evaluation clinic and, when compared to structured functional capacity surveys, if smCPET could better discriminate low functional capacity (≤4.6 metabolic equivalents [METs]).

**Methods:**

After institutional approval, 43 participants presenting for noncardiac surgery who met the following inclusion criteria were enrolled: aged 60 years and older, a Revised Cardiac Risk Index of ≤2, and self-reported METs of ≥4.6 (self-endorsed ability to climb 2 flights of stairs). Subjective METs assessments, Duke Activity Status Index (DASI) surveys, and a 6-minute smCPET trial were conducted. The primary end points were (1) operational efficiency, based on the time of the experimental session being ≤20 minutes; (2) modified Borg survey of perceived exertion, with a score of ≤7 indicating no more than moderate exertion; (3) high participant satisfaction with smCPET task execution, represented as a score of ≥8 (out of 10); and (4) high participant satisfaction with smCPET scheduling, represented as a score of ≥8 (out of 10). Student's *t* test was used to determine the significance of the secondary end points. Correlation between comparable structured surveys and smCPET measurements was assessed using the Pearson correlation coefficient. A Bland-Altman analysis was used to assess agreement between the methods.

**Results:**

The mean session time was 16.9 (SD 6.8) minutes. The mean posttest modified Borg survey score was 5.35 (SD 1.8). The median patient satisfaction (on a scale of 1=worst to 10=best) was 10 (IQR 10-10) for scheduling and 10 (IQR 9-10) for task execution. Subjective METs were higher when compared to smCPET equivalents (extrapolated peak METs; mean 7.6, SD 2.0 vs mean 6.7, SD 1.8; *t*_42_=2.1; *P*<.001). DASI-estimated peak METs were higher when compared to smCPET peak METs (mean 8.8, SD 1.2 vs mean 6.7, SD 1.8; *t*_42_=7.2; *P*<.001). DASI-estimated peak oxygen uptake was higher than smCPET peak oxygen uptake (mean 30.9, SD 4.3 mL kg^–1^ min^–1^ vs mean 23.6, SD 6.5 mL kg^–1^ min^–1^; *t*_42_=7.2; *P*<.001).

**Conclusions:**

Implementation of smCPET in a presurgical evaluation clinic is both patient centered and clinically feasible. Brief smCPET measures, supportive of published reports regarding low sensitivity of provider-driven or structured survey measures for low functional capacity, were lower than those from structured surveys. Future studies will analyze the prediction of perioperative complications and cost-effectiveness.

**Trial Registration:**

ClinicalTrials.gov NCT05743673; https://clinicaltrials.gov/study/NCT05743673

## Introduction

### Background

Assessment of functional capacity or exercise tolerance, as measured by self-reported metabolic equivalents (METs), remains a cornerstone of preoperative risk stratification. METs are defined as multiples of the basal metabolic rate (1 MET=3.5 mL kg^–1^ min^–1^), and self-reported ability to climb 1 flight of stairs has a general consensus of 4 METs [1]. A threshold of ≤4.6 METs (self-reported inability to climb 2 flights of stairs) has been associated with major adverse cardiac events, all-cause mortality, and increased perioperative complications [2-4]. Despite its importance, published reports have cast doubt on the accuracy of provider-driven and self-reported assessment of functional capacity [5,6]. Thus, reliable and efficient methods to precisely characterize functional capacity continue to be of importance in preoperative risk stratification.

Cardiopulmonary exercise testing (CPET) precisely characterizes exercise tolerance by analyzing cellular respiration at rest and during exercise challenges. By measuring resting gas exchange followed by maximal exercise to expose pathophysiological impairments, CPET exploits a symptom-limited approach with a 3-minute resting stage, 3 minutes of unloaded cycling, and a 10- to 12-minute ramp stage with increasing resistance until terminated by the participant [7]. Abnormal CPET measures have been frequently associated with perioperative morbidity, with a peak oxygen uptake (VO_2_) of <15 mL kg^–1^ min^–1^ reported as a threshold for elevated cardiopulmonary risk after thoracic and major noncardiac surgery [8-12]. In addition, peak VO_2_ impairment predicts an increased risk of surgical site infection, postoperative respiratory failure, and critical care readmission [13]. However, CPET has not been widely adopted in preoperative testing, likely due to limited availability, required technical skills, necessity of maximal patient effort, complexity of task, and cost. Yet, conventional preoperative care, usually comprised of subjective or structured, survey-based, clinician estimation of preoperative functional capacity, has demonstrated poor sensitivity in the identification of patients with low functional capacity (≤4 METs), when compared to CPET [13,14].

In contrast to a conventional symptom-limited approach, submaximal cardiopulmonary exercise testing (smCPET) uses a time-limited approach and predictive analytics to provide estimates of peak cardiopulmonary performance [7]. A maximal exercise effort is not required since it analyzes the VO_2_ efficiency slope to predict peak cardiopulmonary performance [15-17]. Of note, the VO_2_ efficiency slope has a strong correlation with peak VO_2_ (*r*=0.941), permitting effort-independent prediction of conventional CPET measures [16]. Brief smCPET has demonstrated diagnostic utility in predicting postoperative length of stay, complications, and prognosis in heart failure, pulmonary hypertension, and other conditions [18-23].

### Objectives

These advantages suggest that time-limited smCPET may be useful for rapid preoperative assessment of exercise tolerance. Therefore, the primary objective was to determine the logistic feasibility of smCPET integration within a high-volume presurgical evaluation clinic. Our measured feasibility end points were (1) operational efficiency, based on the experimental session length being <20 minutes; (2) modified Borg survey of perceived exertion, with a score of ≤7 indicating no more than moderate exertion; (3) high participant satisfaction with smCPET task execution, with a score of >8 (out of 10); and (4) high patient satisfaction with smCPET scheduling, with a score of >8 (of 10). Our secondary objective was to determine if comparable smCPET measures were significantly different from structured survey findings. The secondary end points were a comparison of (1) self-reported subjective METs from a survey versus smCPET equivalents (extrapolated peak METs), (2) Duke Activity Status Index (DASI) [24] estimates versus smCPET equivalents (extrapolated peak METs), and (3) estimated DASI maximal oxygen consumption (estimated peak VO_2_) versus smCPET equivalents (extrapolated peak VO_2_). This study hypothesized that brief smCPET would achieve two objectives: first, meet feasibility end points indicating successful implementation, and second, similar to prior published reports regarding provider-driven functional capacity assessments, identify lower peak METs and VO_2_, when compared to structured surveys.

## Methods

### Trial Design

This is an ongoing prospective open-label clinical device study approved by the Yale University Institutional Review Board (IRB#2000033885; ClinicalTrials.gov: #NCT05743673 [25]; principal investigator: ZJC; date of registration: December 5, 2023). This clinical trial was registered prior to participant enrollment.

### Study Population

Inclusion criteria for study enrollment included age of 60 years and older, a Revised Cardiac Risk Index (RCRI) [26] of ≤2, self-endorsed subjective METs of ≥4 (endorses reliably climbing 2 flights of stairs), and presenting for noncardiac surgery. The aim was to recruit 40 participants for the feThis number was estimated to be adequate to identify any study-related logistic process problems or patient-centered outcome deficiencies and to determine the operational efficiency of this novel system process. The RCRI≤2 criterion was selected given the novelty of smCPET in preoperative evaluation.

Given that participants were screened prior to surgical procedures, exclusion criteria were adapted to maintain current standard-of-care practices in preoperative evaluation, which includes mandatory subspecialty evaluation of select cardiopulmonary conditions. Participants with recorded severe or critical heart valve disease, active exertional angina, nonambulation, gait abnormalities, end-stage renal disease, severe peripheral vascular disease, and neurological motor deficits were excluded. Additionally, non–English-speaking participants, those under legal guardianship, and participants documented to not have personal health care decision-making capacity were also excluded. After prescreening, a phone call was placed by a study team member, and eligible participants were invited for in-person written informed consent, preoperative evaluation, questionnaire assessment of METs, and a 6-minute smCPET experimental session.

### Testing Environment

Testing was performed at the presurgical evaluation (PSE) clinic at Yale New Haven Hospital, which is responsible for more than 40,000 preoperative evaluations per year. On a daily basis, the PSE clinic is staffed by an anesthesiologist, 2 resident physicians, 3 certified nurse practitioners, and 6 nursing staff and contains 6 exam rooms.

### Study Apparatus

**Figure 1:**
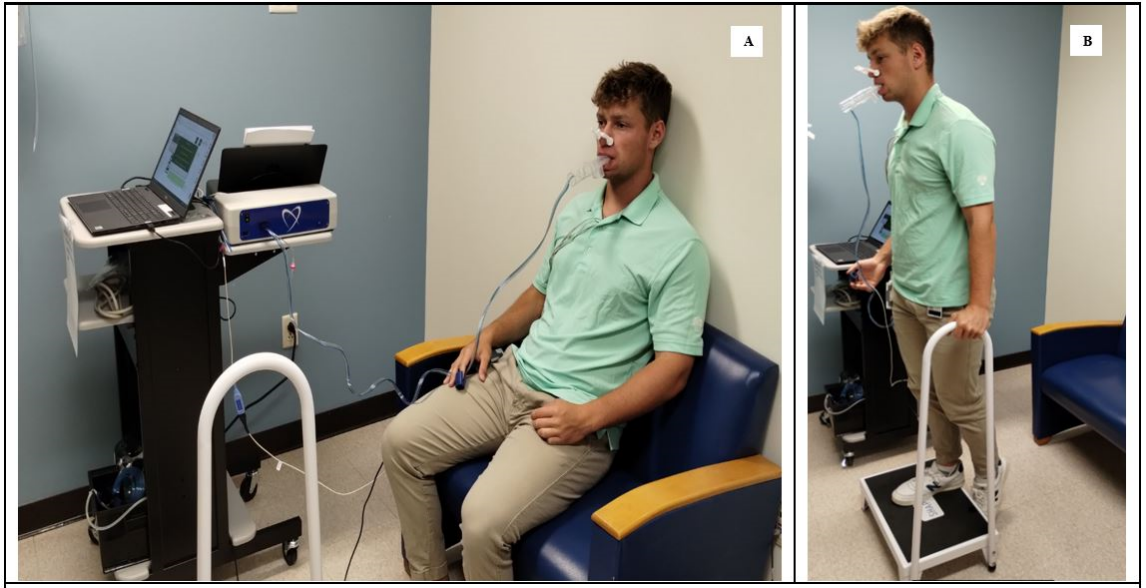
Performance of submaximal cardiopulmonary exercise testing requires (A) 2 minutes of calibration data in the seated position with a disposal mouthpiece connected to the device, (B) 3 minutes of graded exercise using a stair step, and 1 minute of recovery data in the seated position. The submaximal cardiopulmonary device (white and blue box) is visible on the cart, attached to a laptop with calculation software. Coauthor JF gave express permission for the use of his likeness in this simulated participant session.

The US Food and Drug Administration–approved Shape II is a compact, cardiopulmonary, breath-by-breath, exercise testing system that uses brief submaximal exercise effort (3 minutes) to generate multiple quantitative measures of actual and predicted peak cardiopulmonary performance measurements (Figure 1). Predicted peak exercise values are automatically calculated by the device using oxygen efficiency slope equations [16,17]. Furthermore, the device has been previously validated to conventional CPET measurements [27,28]. The compact design allows all the necessary equipment to be placed on a standard rolling cart and was deployed in a PSE clinic examination room (2.4 × 2.4 m). A stairstep (14-cm height) was used for the graded exercise portion. The graded exercise was performed with a device prompt (“begin exercise”), with auditory prompts at 1-minute intervals to increase step frequency if possible. A metronome is used to provide cadence. The device provides an option for either timed or symptom-limited assessment. The timed session was selected for all participants. The timed device session requires a total of 6 minutes: 2 minutes of seated baseline resting data, 3 minutes of escalating exercise using the stairstep, and 1 minute of seated recovery data to generate a variety of individual measures of cardiac and pulmonary physiological data (Multimedia Appendix 1).

### Data Collection

Participants received height, weight, and vital sign measurements (heart rate, blood pressure, and pulse oximetry). Informed written consent was performed, and participants were instructed on smCPET (approximately 5 minutes).

Session time was measured from the beginning of pretest METs questionnaires until the termination of the smCPET recovery phase. A session time of ≤20 minutes would indicate that 24 high-risk participants could be screened per day per machine, permitting high-volume assessment. Session components included (1) a 7-question subjective METs assessment, (2) a 12-question DASI survey, and (3) a timed smCPET (6 minutes).

The modified Borg survey of perceived exertion was performed at session termination. After study interventions, a standard preoperative evaluation was completed, and the participant was discharged. A 24-hour postexperiment survey of minor and major complications and patient satisfaction was performed by telephone (Multimedia Appendix 2). With the exception of the patient satisfaction survey, all survey instruments were adapted from prior publications [29-31]. DASI-estimated peak METs and peak VO_2_ were calculated from individual participants’ DASI scores using the recommended formula.

### Statistical Analysis

End points were reported as continuous variables, described as mean (SD); ordinal variables, as median (IQR and range); and categorical variables, as number (%). Secondary end points were first analyzed using the Student *t* test (2-tailed) to compare differences in comparable measurements. Agreement between structured survey findings and smCPET comparable measurements was assessed using 2 approaches. First, a Pearson correlation coefficient was calculated to evaluate the strength and direction of the linear relationship, followed by a Bland-Altman analysis to assess agreement between methods, where differences between paired measurements were plotted against their means. Mean difference (MD) and 95% limits of agreement (LOAs) were calculated. All analyses were carried out on R (version 4.1.1; R Foundation for Statistical Computing). To reduce the introduction of bias, a complete case analysis for missing data was performed, where participants with missing data were excluded from the analysis of the respective end point. Similarly, dropouts were removed from the analysis. A *P* value of <.05 was accepted for significance.

### Ethical Considerations

This study was performed in accordance with the principles of the Declaration of Helsinki. Approval was granted by the Yale University Institutional Review Board (IRB#2000033885). Informed consent was obtained from all participants included in the study. All provided data were deidentified prior to analysis to maintain participant privacy. No monetary compensation was provided to the participants. JF has given express written informed consent for the publication of his image in Figure 1.

## Results

### Participant Recruitment

**Figure 2:**
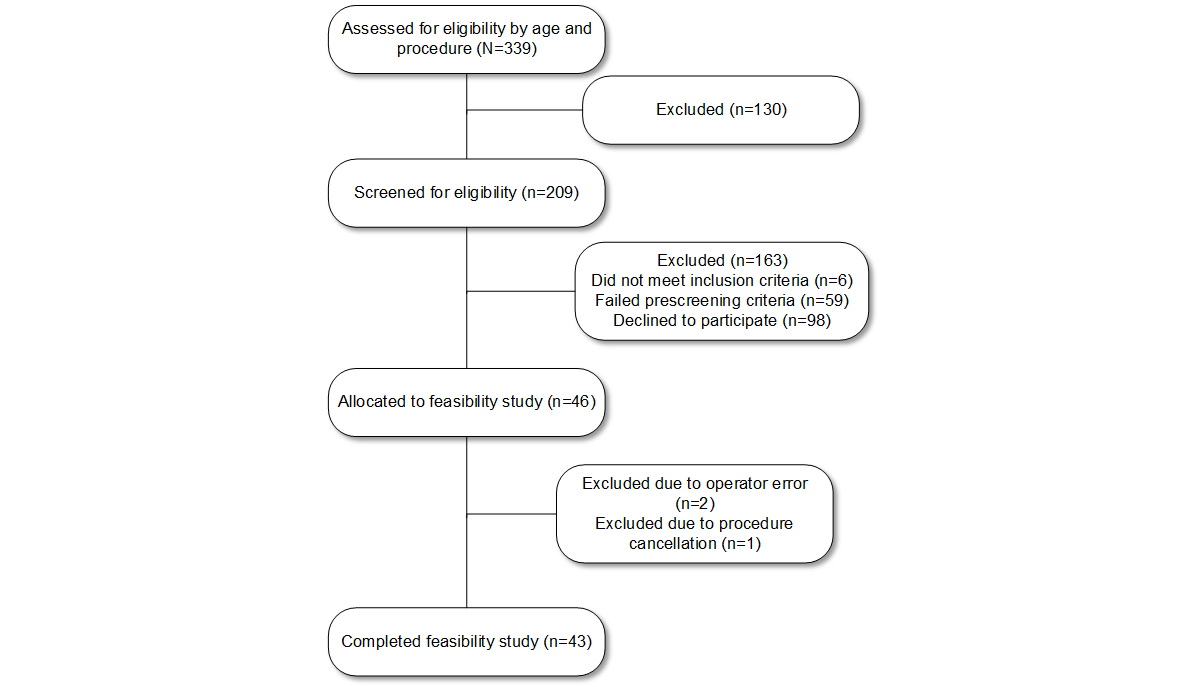
A flow diagram of participant enrollment.

We identified 209 (61.6%) out of 339 potential participants that met eligibility criteria; 6 (1.8%) did not meet the inclusion criteria, 59 (17.4%) failed the prescreening criteria, and 98 (28.9%) declined study participation (Figure 2). Initially, 46 participants were enrolled but 3 (7%) were excluded (operator error: n=2; surgery cancellation: n=1), for a final cohort of 43 participants.

### Baseline Characteristics

Trial participants had a median age of 68 (IQR 66-73, range: 60-86 years), 20 (47%) of 43 were female, and the mean BMI was 27.5 (SD 6.0) kg/m^2^. Preoperative RCRI score was a median of 1 (IQR 1-1; range 1-2). Essential hypertension (22/43, 51%), hyperlipidemia (17/43, 39%), and solid tumor (25/43, 58%) were the most common premorbid conditions. A total of 22 (51%) out of 43 participants were former or active smokers. Major abdominal surgeries comprised 27 (63%) out of the 43 surgical procedures (Table 1).

All participants completed the smCPET session components. The mean peak respiratory exchange ratio was 0.88 (SD 0.12), consistent with submaximal effort (respiratory exchange ratio<1.05). The ventilatory threshold was achieved in 22 (51%) of 43 participants (mean 227.9, SD 21.9 seconds in those that achieved ventilatory threshold).

**Table 1:** Baseline demographical data of the study cohort (n=43).

Variable				Values
Age (years), median (IQR; range)				68 (66-73; 60-86)
**Sex, n (%)**				
	Male			23 (54)
	Female			20 (47)
BMI (kg/m^2^), mean (SD)				27.5 (6.0)
Revised Cardiac Risk Index score, median (IQR; range)				1 (1-1; 1-2)
**Preoperative comorbidities, n (%)**				
	Essential hypertension			22 (51)
	Hyperlipidemia			17 (40)
	Ventricular dysrhythmia			1 (2)
	Congestive heart failure			1 (2)
	Myocardial infarction			3 (7)
	Cerebrovascular disease			1 (2)
	Chronic obstructive pulmonary disease			3 (7)
	Asthma			4 (9)
	Obstructive sleep apnea			3 (7)
	History of prior lung resection			1 (2)
	Diabetes mellitus			7 (16)
	Thyroid disorders			7 (16)
	Solid tumor			25 (58)
	Anemia			1 (2)
**Social history, n (%)**				
	Smoking			
		Ever		22 (51)
			Active	4 (9)
			Former	18 (42)
		Never		21 (49)
	Marijuana use (active)			4 (9)
	Alcohol use			
		Active		24 (56)
		Former		16 (37)
		Never		3 (7)
**Cardiovascular medication use, n (%)**				
	Beta-blocker			14 (33)
	Calcium channel antagonist			9 (21)
	ACE/ARB^a^ antagonist			16 (37)
	Diuretic			12 (28)
**Surgical categories, n (%)**				
	Abdominal major			27 (63)
	Musculoskeletal major			4 (9)
	Neurosurgical major			2 (5)
	Thoracic major			5 (12)
	Other major			5 (12)

^a^ACE/ARB; angiotensin converting enzyme inhibitor/angiotensin receptor blockers.

### Primary End Points

The mean experimental session time was 16.9 (SD 6.8) minutes. The modified Borg survey score after experimental sessions was mean 5.35 (SD 1.8), corresponding to moderate perceived exertion. All 43 participants were reached for the 24-hour postexperiment survey. The median patient satisfaction (on a scale of 1=worst to 10=best) was 10 (IQR 10-10) for scheduling and 10 (IQR 9-10) for task execution. No major or minor complications associated with study testing were reported by participants. Operational efficiency was achieved within 15 experimental sessions among 4 study team members (3 physicians and 1 undergraduate researcher).

### Secondary End Points

**Figure 3:**
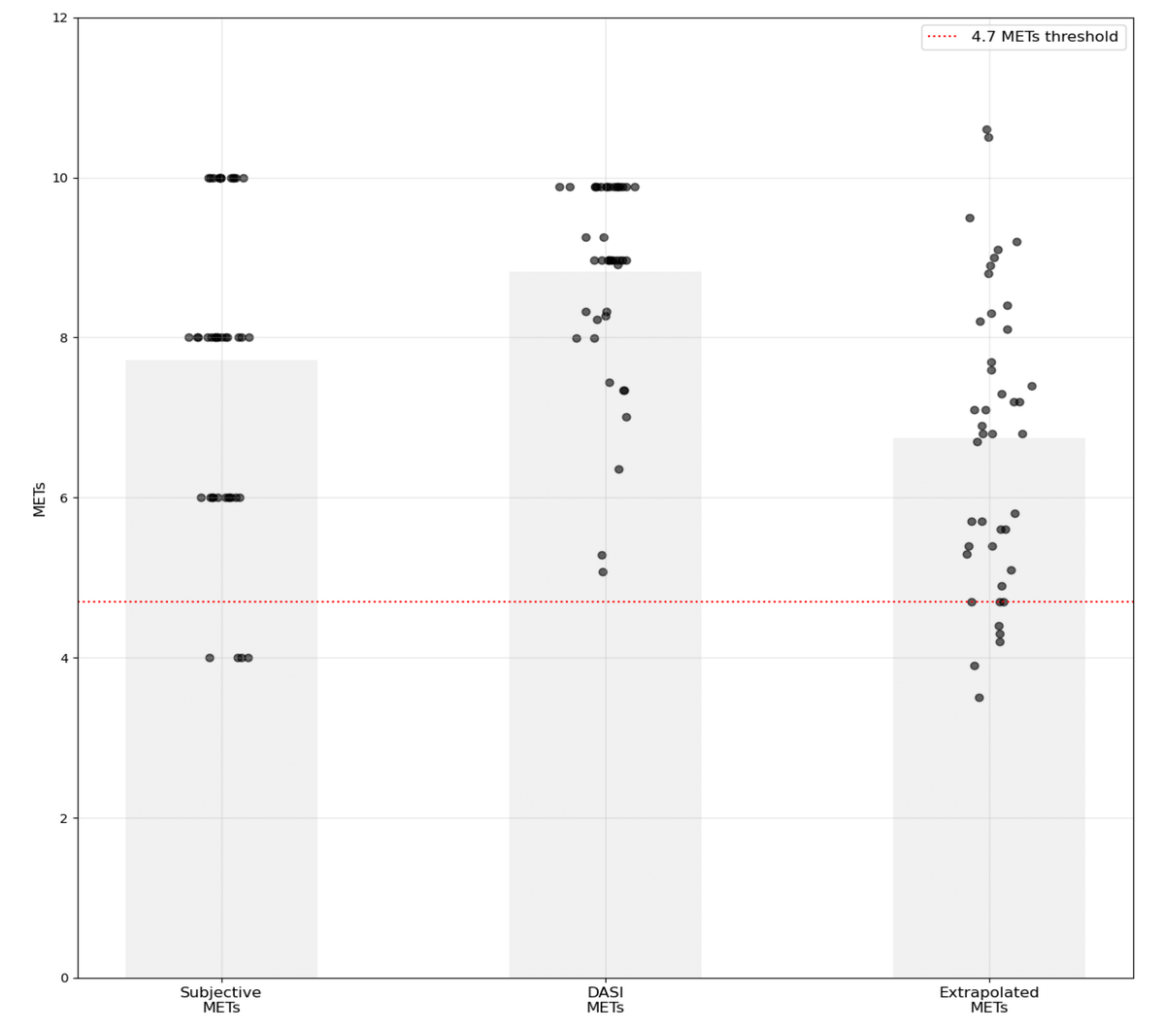
Comparison of elicited METs from 2 structured survey instruments (subjective METs and DASI) compared to predicted peak METs from submaximal cardiopulmonary exercise testing (dotted line represents 4.7 METs). DASI: Duke Activity Status Index; MET: metabolic equivalent.

Average self-reported peak METs were higher when compared to smCPET equivalents (extrapolated peak METs; mean 7.6, SD 2.0 vs mean 6.7, SD 1.8; t_42_=2.1; *P*<.001). DASI-estimated peak METs were higher when compared to the smCPET equivalents (extrapolated peak METs; mean 8.8, SD 1.2 vs mean 6.7, SD 1.8; t_42_=7.2; *P*<.001). DASI-estimated peak VO_2_ was higher than the smCPET equivalent (extrapolated peak VO_2_; mean 30.9, SD 4.3 mL kg^–1^ min^–1^ vs mean 23.6, SD 6.5 mL kg^–1^ min^–1^; t_42_=2.1; *P*<.001). Figure 3 provides a comparison of values obtained from smCPET compared to structured survey–estimated peak METs and DASI-estimated peak METs.

To analyze the congruency between the 3 study instruments, correlation and Bland-Altman analyses were performed. DASI-estimated METs showed a moderate positive correlation versus subjective METs (*r*=0.63; *P*<.001). Weaker correlations were observed with smCPET-derived extrapolated peak METs versus DASI and subjective METs (*r*=0.29; *P*=.06 and *r*=0.144; *P*=.36, respectively). DASI versus subjective METs showed an MD of 1.1 (SD 1.49; 95% LOAs –1.82 to 4.02) METs, while DASI versus smCPET-derived extrapolated peak METs showed larger discrepancies with an MD of 2.07 (SD 1.86; 95% LOAs –1.58 to 5.73) METs. The comparison between subjective METs and smCPET-derived extrapolated peak METs showed intermediate systematic bias with the widest LOAs (MD 0.97, SD 2.43 METs; 95% LOAs –3.80 to 5.75). When comparing DASI and smCPET-derived extrapolated peak VO_2_ values, a positive MD was observed, indicating that DASI estimates were consistently higher (MD 7.23, SD 6.54 mL kg^–1^ min^–1^; 95% LOA –8.11 to 21.12) and showed poor agreement (*r*=0.28; Multimedia Appendix 3).

## Discussion

### Principal Findings

Integration of brief smCPET in a high-volume PSE clinic was feasible as measured by the primary end points of session time, patient satisfaction with smCPET task execution, perceived exertion, and session scheduling. The operational efficiency of study team members was acceptable within 15 experimental sessions. Finally, smCPET measures of peak METs and VO_2_ were significantly lower, when compared to comparable structured survey results.

Mean session time, which included the subjective METs survey, DASI, and 6-minute smCPET session, was 16.9 (SD 6.8) minutes, with progressive improvement over the study time period as operators (n=4) became facile with the study instrument (Multimedia Appendix 4). It is important to note that smCPET comprised 6 minutes of the session time, shorter than reported times with conventional CPET (15-20 min/session) [32]. In high-volume PSE, this may be advantageous, as patients are often seen on short notice for preoperative evaluation. Participants were able to flexibly arrange smCPET around other clinic appointments, decreasing study participants’ time constraints. This likely enhanced our high satisfaction score for scheduling. High patient satisfaction was observed with task execution and perceived exertion during smCPET. The tested device uses a stationary stairstep for graded exercise, which was frequently familiar to participants. The short duration of graded exercise (3 minutes) was not perceived by any participant as maximum exertion by the Borg survey, likely contributing to the high level of patient satisfaction. Second, the Borg score of <7 after smCPET suggests a reasonable probability of success when transitioning its use to patients with more severe comorbidities, or preoperative deconditioning. It is important to note that the ventilatory threshold, or anaerobic threshold, was not measurable in 50% of our cohort, suggesting that the brief graded exercise contributed to the reported exertion level and high participant satisfaction.

One of the goals of smCPET is to make precise cardiopulmonary evaluation more widely available and patient centered, advantages that are acknowledged by its increasing adoption in the routine assessment of heart failure and pulmonary hypertension. Consistent with large-scale CPET application in cardiovascular clinical trials, smCPET did not result in findings of major or minor complications despite encouraging participants to safely provide their best effort within the timed and graded exercise component [33]. This is reassuring, as early termination of preoperative CPET trials, due to participant fatigue, safety, or other considerations, has been reported to be approximately 11% [13]. However, we purposefully selected functionally independent participants with self-reported ≥4.6 METs, and expansion to patients who are less functionally independent may result in higher smCPET session failure rates. Regardless, the safety of smCPET has been suggested by its routine application to high-risk and frail populations with severe cardiopulmonary disease, suggesting that a wide spectrum of preoperative populations can be safely tested using smCPET [20,22,34].

The structured survey estimated METs were, on average, significantly higher than their smCPET equivalents. Using the subjective METs structured survey, 8 (19%) of 43 participants reported peak METs within 10% of smCPET extrapolated peak METs, 12 (28%) were underestimated by >10%, and 23 (53%) were overestimated by >10%, when compared to smCPET values. Brief smCPET identified that 8 (19%) out of 43 study participants had ≤4.6 extrapolated peak METs (peak VO_2_ equivalent: 14 mL kg^–1^ min^–1^), corresponding to a METs threshold associated with higher perioperative cardiovascular risk [1,4]. Furthermore, smCPET identified 9 (21%) out of 43 participants with an age-adjusted peak VO_2_ of less than 20 mL kg^–1^ min^–1^, corresponding to poor aerobic capacity, and 2 (5%) with an extrapolated peak VO_2_ less than 15 mL kg^–1^ min^–1^, a measure frequently associated with higher perioperative risk [35]. These findings support prior descriptions of provider-driven and structured survey overestimation bias, highlighting the challenge of obtaining an accurate preoperative functional capacity assessment. Clinicians, when compared to CPET, had a 19.2% sensitivity in identifying low functional capacity (≤4 METs) [13,36]. Other investigations have also observed that preanesthesia evaluation calculation of self-reported METs overestimate functional capacity when compared to CPET assessment [6]. DASI was also found to poorly predict participants with lower peak VO_2_ [13,24,36]. In a cohort of participants that would not necessarily receive extensive preoperative assessment, given that 100% reported the ability to reliably climb 2 flights of stairs, this may suggest opportunities to identify and preemptively optimize unexpected cardiopulmonary impairments prior to surgical intervention.

Worldwide, value-based health care has been a significant priority, and conventional preoperative evaluation may increase overall testing costs without improving perioperative outcomes [37-39]. Implementing brief smCPET for individualized preoperative cardiovascular evaluation may improve the precision of preoperative cardiovascular risk assessment and may potentially curb excess preoperative cardiovascular testing commonly associated with older age and patients with higher comorbidities [40-42]. However widespread adoption of this technology in the perioperative space will require (1) further evidence of smCPET predictive validity for perioperative outcomes, (2) characterization of optimal system processes for patient selection, and (3) justification of cost-benefit.

### Study Limitations

Several study limitations limit generalizability to other populations. Selection bias should be acknowledged given that participants who volunteered for the study are likely to be more health-conscious than usual patients who undergo PSE. A measurement bias may be introduced into the study given that researchers may unconsciously influence participant performance on smCPET or interpret results differently based on unconscious expectations. Similarly, a recall bias is often introduced when using structured, interview-style questionnaires such as those used in our study. Instrument bias may similarly impact smCPET findings; however, this is substantially reduced by routine device calibration.

Confounding factors are similar, where participants with higher fitness levels would find it easier to adapt to the stairstep exercise challenge. Our inclusion criteria purposely selected participants with lower comorbidities to ensure successful participation rates for this feasibility study. We acknowledge that certain premorbid conditions and chronic medication usage may influence smCPET participants’ performance, but we did not balance this factor in this exploratory study. Although CPET and smCPET predictive performance with cardiovascular perioperative morbidity and mortality has been previously published, our cohort is not yet powered for the assessment of perioperative outcomes with this device [19,23,43,44]. Finally, the finding of no device-related adverse events should be cautiously interpreted given the small sample size and the possibility of rare exercise-induced adverse events.

### Conclusions

In summary, we observed that smCPET implementation was well accepted into the workflow of a high-volume PSE clinic. Operator efficiency with the smCPET instrument was rapid and achieved relative parity at 15 participant sessions. smCPET, when compared to usual session times for conventional CPET of 15-20 minutes, uses less than half the time (6 minutes), making it attractive for the purposes of precise but time-efficient preoperative evaluation of exercise tolerance. This feasibility analysis has (1) reinforced the operational integrity of our active study protocol assessing smCPET findings with perioperative outcomes and (2) affirmed satisfactory patient-centered outcomes with study procedures. Studies should further expand smCPET predictive validity to postoperative cardiopulmonary complications, assess cost-effectiveness, and develop optimal system processes for patient selection.

## Appendix

Multimedia Appendix 1 A detailed description of the submaximal cardiopulmonary exercise testing device and its measures.

Multimedia Appendix 2 Adapted subjective METs survey questions and 24 hour postsession minor and major adverse events survey. MET: metabolic equivalent.

Multimedia Appendix 3 Bland-Altman plots for the compared measures: (A) subjective METs versus smCPET-extrapolated peak METs showed a mean difference of 0.97 METs but the widest LOAs; (B) DASI–estimated peak METs versus smCPET-extrapolated peak METs showed a mean difference of 2.07 METs, the largest discrepancy; (C) DASI-estimated peak METs versus subjective METs showed a mean difference of 1.1 METs but narrower LOAs; and (D) DASI-estimated peak VO2 versus smCPET-extrapolated peak VO2 showed that DASI had consistently higher estimates, with a mean difference of 6.5 mL kg–1 min–1. DASI: Duke Activity Status Index; LOA: limit of agreement; mean diff: mean difference; MET: metabolic equivalent; smCPET: submaximal cardiopulmonary exercise testing; VO2: peak oxygen uptake.

Multimedia Appendix 4 Operator efficiency as a function of session time (y-axis), defined as performance of two structured functional capacity survey instruments and submaximal cardiopulmonary exercise testing, versus session number (x-axis).

## Abbreviations

CPET: cardiopulmonary exercise testing
DASI: Duke Activity Status Index
LOA: limit of agreement
MD: mean difference
MET: metabolic equivalent
PSE: presurgical evaluation
RCRI: Revised Cardiac Risk Index
smCPET: submaximal cardiopulmonary exercise testing
VO_2_: oxygen uptake

## Data Availability

The datasets generated or analyzed during this study are available from the corresponding author on reasonable request.
