# Supplementary material for: Implementation of Brief Submaximal Cardiopulmonary Testing in a High-Volume Presurgical Evaluation Clinic: Feasibility Cohort Study
Source: JMIR Perioper Med. 2025 Feb 17;8:e65805. doi: 10.2196/65805 (PMC11888076; doi:10.2196/65805)
Supplement: Multimedia Appendix 1 [file periop_v8i1e65805_app1.docx]

| **Multimedia Appendix 1. Selected Measurements of Submaximal Cardiopulmonary Exercise Testing.** | | |
| --- | --- | --- |
| **Variable** | **Description** | **Commentary** |
| **Summary of Shape II® metabolic analyzer used in study:** The device uses breath by breath sampling during calibration and exercise challenge using a differential pressure pneumotach method for volume calibration and measurement, an infrared sensor for CO_2_ and a paramagnetic sensor for O_2_ measurements. Automated calibration using a calibration gas mixture (15.6% O_2_/5% CO_2_) is performed at regular intervals. The Shape II calculations used for causes of exertional dyspnea differentiation are Artificial Intelligence (AI) based algorithms previously calibrated and validated against conventional cardiopulmonary exercise testing methods.  **Summary of the Oxygen Utilization Efficiency Slope (OUES)**: The OUES equation forms the foundation for the prediction of peak extrapolated oxygen uptake (VO_2_) with submaximal cardiopulmonary exercise testing. The OUES represents the relationship between oxygen uptake (VO_2_) and minute ventilation (VE) during escalating exercise challenge. It is expressed as the slope of the linear relationship between VO_2_ and the logarithm of VE. OUES is calculated using the following equation: VO_2_ = a x log(VE) + b, where a =OUES and b =y-intercept of the regression line (oxygen uptake when minute ventilation is equal to 1 liter/min, reflecting baseline oxygen consumption). To derive peak VO_2_ values using OUES, the following steps are performed; plot VO_2_ against logVE through the exercise challenge, calculate the slope of this relationship using linear regression and extrapolate to predict maximal ventilation and estimate peak VO_2_. | | |
| HR (resting) | Resting heart rate | Measured during 1st stage prior to exercise. |
| HR (peak) | Peak heart rate achieved during exercise | Measured during 2nd stage during exercise. |
| % HR reserve utilized | Percentage of heart rate reserve utilized | Percentage of resting heart rate and the age-dependent predicted maximum heart rate |
| % HR max predicted attained | Percentage of predicted maximum achieved heart rate achieved | Difference between maximum measured heart rate during exercise and age-dependent estimated maximum heart rate. |
| CRI | Chronotropic Recovery Index | A measure of heart rate recovery after exercise |
| RR rest | Respiratory rate at rest | Measured during 1st stage prior to exercise. |
| RR end exercise | End-exercise respiratory rate | Measured during 2nd stage during exercise. |
| End-tidal CO_2_ (rest) | End-tidal carbon dioxide, at rest | Influenced by cardiac output, pulmonary vascular resistance, and chronic hypoventilation syndromes |
| End-tidal CO_2_ (peak) | End-tidal carbon dioxide, peak exercise | Peak carbon dioxide during exercise |
| Resting SpO_2_ | Resting pulse oximetry | Resting peripheral oxygenation |
| Peak SpO_2_ | Peak exercise pulse oximetry | Peak exercise peripheral oxygenation |
| RER | Respiratory Exchange Ratio | Ratio between metabolic production of CO_2_ and uptake of O_2_ |
| VE/VCO_2_ slope | Minute ventilation/CO2 production slope | Breathing efficiency slope reflects the efficiency of elimination of CO_2_ |
| Δ EtCO_2_ (rest to end exercise) | Change in end-tidal carbon dioxide during exercise | A measure of cardiac output and pulmonary blood flow |
| Gxcap (peak) | Gas exchange-derived pulmonary vascular capacitance at peak exercise | Shown to correlate with cardiac output and inversely with pulmonary vascular resistance (PVR) and DLCO; in patients with PAH or PVH, Gxcap is reduced due to increased PVR. |
| OUES (linear slope) | Oxygen uptake efficiency slope, percentage of expected | Assesses how well oxygen is extracted from the inhaled air, distributed to the muscles by the cardiopulmonary system, and utilized by the muscles in energy metabolism; Particularly valuable in assessing exercise capacity and response to medications or rehabilitation |
| Peak Attained METs | Metabolic Equivalents | Metabolic equivalents attained during submaximal exercise. |
| Peak Extrapolated METs | Metabolic Equivalents | Peak estimated metabolic equivalents |
| O_2_ pulse | Amount of oxygen consumed per heartbeat |  |
| Sub-maximal VO_2_ (peak attained) | Sub-maximal exercise oxygen uptake | Achieved peak maximal oxygen uptake during submaximal exercise. |
| Extrapolated maximum VO_2_ | Predicted maximal exercise oxygen uptake | Calculated peak maximal oxygen uptake. |
| MVI score | Dimensionless; (cumulative sum score) | Combined cardiopulmonary index with threshold value of normal vs. impairment. |
| Cardiac disease silo score | Dimensionless; (cumulative sum score) | Parameters: VE/VCO_2_ slope, O_2_ pulse to VO_2_ slope, circulatory equivalents, HR recovery |
| Pulmonary Vascular Disease silo score | Dimensionless; (cumulative sum score) | Parameters: VE/VCO_2_ slope, peak Gxcap, resting SpO_2_, SpO_2_ desaturation |
| Chronic Obstructive Pulmonary Disease silo score | Dimensionless; (cumulative sum score) | Parameters: FEV1 % Predicted, breathing reserve, SpO_2_ desaturation, P mixed expired CO_2_/P end-tidal CO_2_ ratio (V/Q ratio) |
| Restrictive Lung Disease silo score | Dimensionless; (cumulative sum score) | Parameters: FVC % Predicted, SpO_2_ desaturation, VT max / VT rest, RR/VCO_2_ slope (lung stiffness) |
| De-conditioning silo score | Dimensionless; (cumulative sum score) | Parameters: extrapolated peak VO_2_, % ideal BMI, HR to VO_2_ linear regression slope, HR recovery 1 minute post exercise |
| V/Q plot | Dimensionless; (cumulative sum score); (Normal, Left ventricular dysfunction, Chronic Obstructive Pulmonary Disease, Transitional or Pulmonary Arterial Hypertension) | Assesses which physiological area may contribute to visualized impairments during submaximal exercise testing. |
| AwCap peak (VT pk x PECO_2_ peak) | airway capacitance; L x mmHg | Airway Capacitance |
| VT pk (peak attained VT) | Peak attained tidal volume in L/min | Value obtained during exercise. |
| VE peak (peak attained VE) | Peak attained minute ventilation in L/min | Value obtained during exercise. |
| Abbreviations: HR; heart rate; CRI; chronotropic Recovery Index; RR; respiratory rate (in breaths/min); CO_2_; carbon dioxide; SpO_2_; pulse oximetry; RER; respiratory Exchange Ratio; Gxcap; pulmonary arterial capacitance; OUES; oxygen uptake efficiency slope; METs; metabolic equivalents; VO_2_; oxygen consumption; MVI; multivariable index; VE; minute ventilation; VT; tidal volume; AwCap; airway capacitance. | | |
