# Supplementary material for: Implementation of Brief Submaximal Cardiopulmonary Testing in a High-Volume Presurgical Evaluation Clinic: Feasibility Cohort Study
Source: JMIR Perioper Med. 2025 Feb 17;8:e65805. doi: 10.2196/65805 (PMC11888076; doi:10.2196/65805)
Supplement: Multimedia Appendix 2 [file periop_v8i1e65805_app2.pdf]

Multimedia Appendix 2.

a. Adapted Self-Reported Subjective Metabolic Equivalents Survey

Question	Estimated Metabolic Equivalents	Accepted value
Can you perform the following activities (yes/no)		
Watching television, writing, desk work?	1-2	2
Walk slowly on level ground (1.7mph)	2-3	3
Climb two flights of stairs, without stopping to rest?	3-5	4
Walk at moderate pace on level ground (3mph/20 minute mile), ride a stationary bicycle at very light intensity or vacuum around the home?	3-5	4
Ride a stationary bicycle at moderate intensity?	5-6	6
Jogging, fast swimming, play soccer or tennis?	7-8	8
Run a 7.5 minute/mile, jump-rope 100 skips/minute, run up the stairs	10-13	10

b. ***24-hour post-experimental survey***

Did you have shortness of breath at rest, dizziness, or fainting (syncope) or lightheadedness (near-syncope)?
Did you have new weakness, fall, or muscle spasm?
Did you have new nausea, emesis or other severe gastrointestinal upset?
Did you have to return to the hospital, emergency room, or your primary care provider for any reason?
How satisfied were you with the experimental session in terms of scheduling? (0-10, 10 is most satisfied)
How satisfied were you with the experimental session in terms of ease of performing the requested tasks? (0-10, 10 is most satisfied)
