# Supplementary figures and images for: Implementation of Brief Submaximal Cardiopulmonary Testing in a High-Volume Presurgical Evaluation Clinic: Feasibility Cohort Study
Source: JMIR Perioper Med. 2025 Feb 17;8:e65805. doi: 10.2196/65805 (PMC11888076; doi:10.2196/65805)

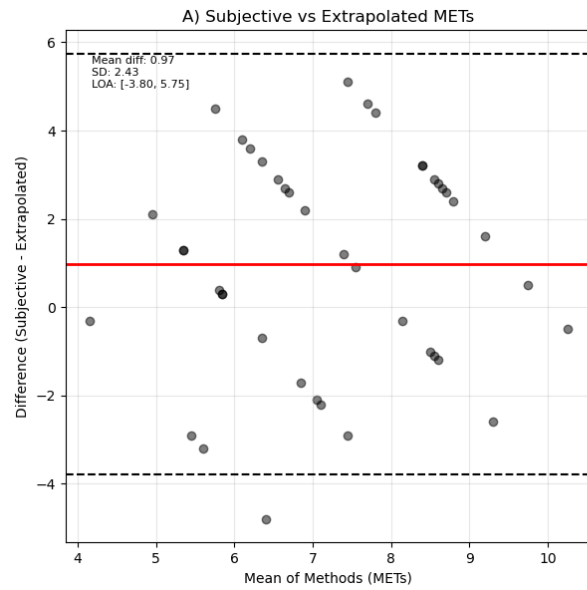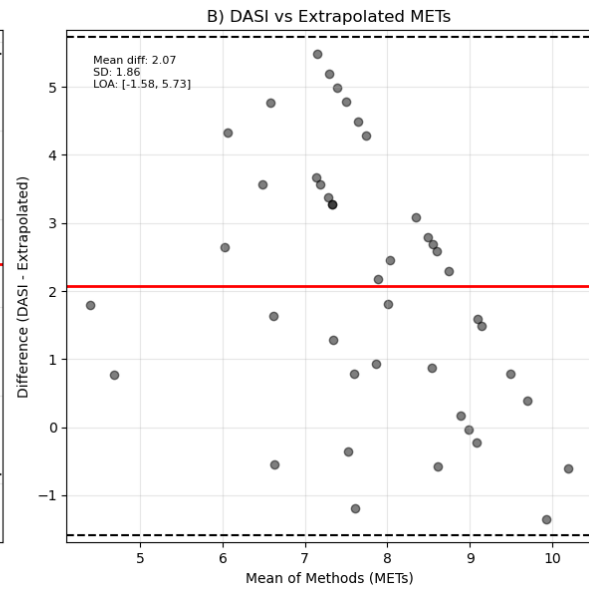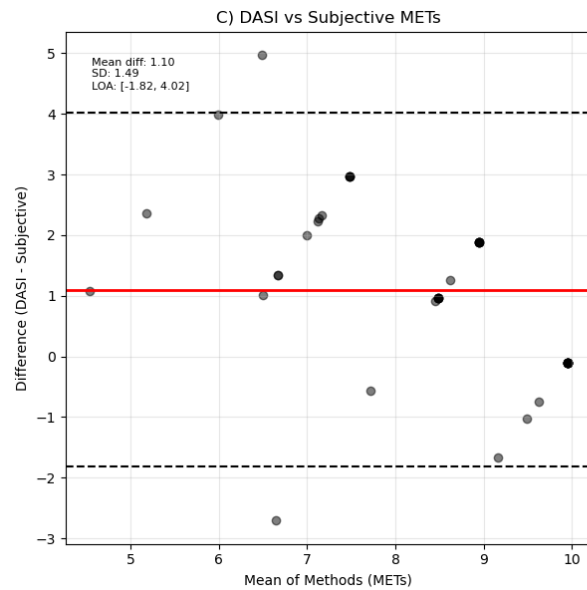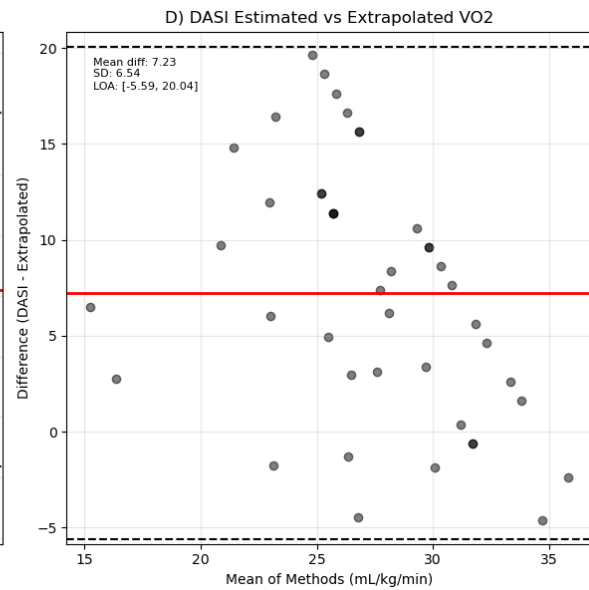

Supplement: Multimedia Appendix 3 [file periop_v8i1e65805_app3.pdf]

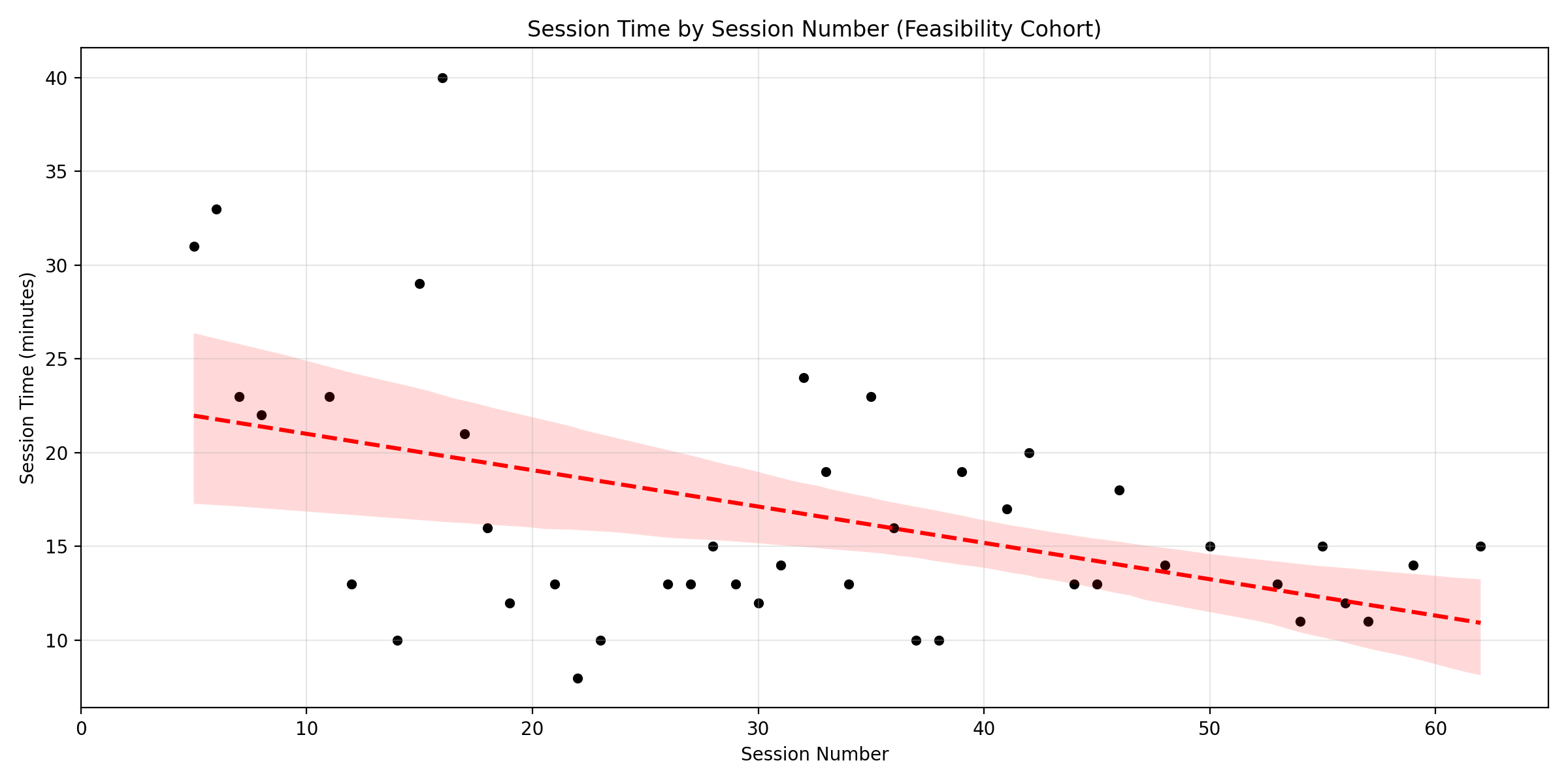

Supplement: Multimedia Appendix 4 [file periop_v8i1e65805_app4.png]
